# Influence of peracetic acid-ethanol sterilisation on the biomechanical properties of human meniscus transplants

**DOI:** 10.1186/s40634-021-00336-z

**Published:** 2021-03-05

**Authors:** Volker Eras, Josefine Graffunder, Norus Ahmed, Jan C. Brune

**Affiliations:** German Institute for Cell and Tissue Replacement (DIZG, gemeinnützige GmbH), Haus 42, Köpenicker Str. 325, 12555 Berlin, Germany

**Keywords:** Meniscus, Allograft, Transplant, Sterilisation, Peracetic acid, Biomechanics

## Abstract

**Purpose:**

Meniscus allograft transplantation (MAT) is a possible treatment for patients suffering with pain after meniscectomy. Here, peracetic acid (PAA) sterilised meniscus transplants were investigated on whether they would provide an adequate alternative to fresh-frozen transplants in their viscoelastic and mechanical properties.

**Methods:**

In this analysis, 31 menisci donors (26 male and 5 female) were included. The average donor age was 49.87 years, ranging from 32 to 65 years. Menisci of matched pairs of knees underwent chemical sterilisation while counterparts were left fresh-frozen. Stiffness and load to failure were determined via suture retention. Further menisci were analysed while attached to the tibial bone block using a novel test device to mimic physiological load distribution. Meniscus relaxation, stiffness and failure loads were determined. Histology and biphasic properties of the menisci were examined and results were analysed using paired t-tests.

**Results:**

A novel custom built test device allowed the application of physiological loads for suture retention testing and revealed no significant differences between PAA sterilised (14.85 ± 4.46 N/mm, 50.49 ± 17.01 N) and fresh-frozen (18.26 ± 4.46 N/mm, 59.49 ± 21.07 N) regarding stiffness and failure load, respectively. Furthermore, initial 200 N loading showed significantly higher strain in sterilised menisci (18.87 ± 1.56) compared to fresh frozen (13.81 ± 1.04). Load relaxation experiments demonstrated significantly lower relaxation for sterilised menisci (77.71 ± 1.62) compared to fresh-frozen (89.11 ± 1.00, *p*-value < 0.0001).

**Conclusion:**

Peracetic acid sterilised human menisci performed equally to fresh-frozen counterparts in a suture retention test and in physiological failure testing providing an adequate alternative. However, meniscus relaxation, biphasic properties and strain were shown to be significantly different between the groups. A common problem of MAT is graft extrusion or shrinkage, therefore the parameters measured here should be considered and may influence meniscus extrusion after transplantation.

**Level of evidence:**

n/a (experimental study)

## Introduction

The meniscus is an essential part of the complex knee mechanics as it acts as a shock absorber by resisting tibiofemoral pressure [19, 27]. It stabilizes the knee, improves congruency and further provides lubrication [17, 34, 52]. Severe meniscal injuries are often treated by partial or total meniscectomy. Mensicus allograft transplantation provides treatment for post-meniscectomy syndrome in the presence of a previous total or sub-total meniscectomy [48]. When left untreated, a meniscectomy is associated with early cartilage damage and in many cases leaving no other option than knee arthroplasty [13, 45]. Especially for young and active patients, who, with todays life expectancy, will undergo several cycles of revision arthroplasty, meniscus transplantation therefore provides a valuable alternative [33, 36, 37, 48].

Currently, three options are available for meniscal replacement such as the collagen meniscus implant (CMI) derived from a bovine collagen [20], Actifit, a polyurethane scaffold [28] and 3D printed scaffolds [16]. Additionally, tendon autografts are also used. However, for meniscus substitution, allografts have been used and historically supplied as fresh-frozen (FF), deep-frozen, cryopreserved or lyophilized grafts [38, 51].

Sterilization of allografts is important to ensure tissue safety and sterility. Two sterilisation methods are widely used to ensure tissue safety, gamma irradiation and ethylene oxide [6, 21, 24, 57]. Gamma irradiation is associated with negative effects on the biomechanical properties of meniscal tissue [6, 15, 49, 56, 57]. Ethylene oxide may induce an inflammatory response [24]. A more recent approach is supercritical CO_2_ which seems to outperform gamma irradiation with regards to biomechanical properties of meniscus transplants [5, 47]. However, validation of its sterility assurance level has not yet been confirmed.

Peracetic acid (PAA) ethanol sterilisation is a validated procedure to reliably eliminate bacterial, fungal and viral contaminations as well as spores [41, 42]. PAA sterilisation did not show an effect on the biomechanics of cortical bone [22]. Nevertheless, PAA sterilised cancellous bone has inferior biomechanical properties but can be successfully vitalized using mesenchymal stromal cells [43, 54].

In 2008, Scheffler and colleagues performed an in vivo sheep study comparing the remodelling and biomechanical behaviour of PAA sterilised and non-sterilised long flexor tendons for anterior cruciate ligament (ACL) reconstruction and analysed at 6 and 12 weeks [50]. The authors demonstrated that the remodelling of PAA-tendons was delayed. Hence, the failure properties of PAA-grafts were inferior to those of FF grafts at the given time points. Two major limitations of that study included the uncontrolled weight bearing of the animals and the selected early time points. In a previous study, the authors did not find any significant differences in the viscoelastic and mechanical properties of bone-patellar tendon-bone (BPTB) allografts when comparing PAA sterilised and fresh-frozen counterparts in vitro [51]. To the authors’ best knowledge, no study has focused on the PAA sterilisation of human meniscus transplants. Therefore, the aim of the present study is to investigate the influence of PAA sterilisation on the viscoelastic and mechanical properties of human meniscus transplants. We hypothesise that PAA sterilisation does not negatively affect the failure properties of meniscus transplants, but might display differences in the viscoelastic properties.

## Material and methods

### Donor tissue and specimen preparation

**Fig. 1:**
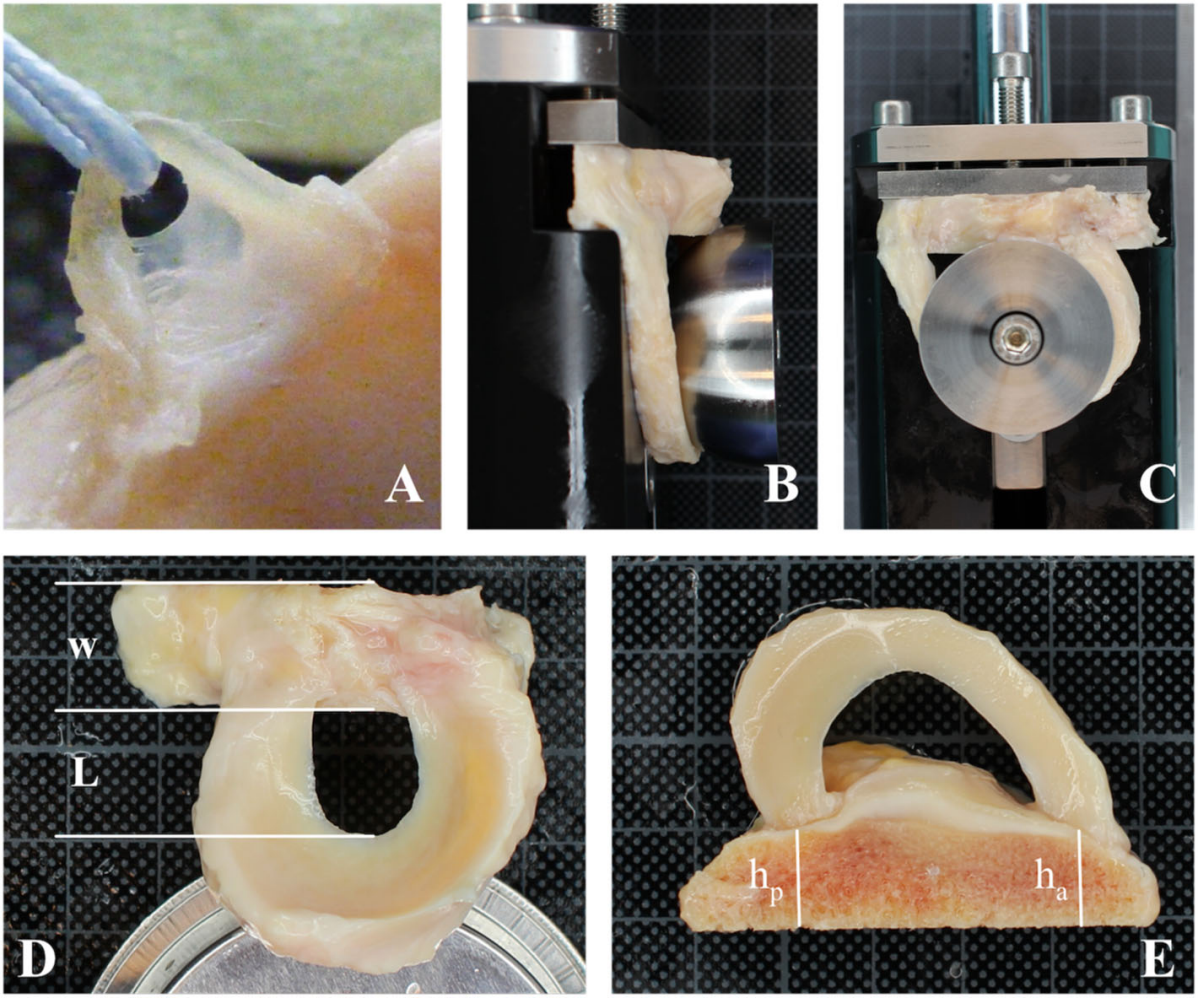
A suture loop (**a**) separates a collagen bundle with a cross section of 0.5 mm^2^. The collagen bundle was channeled through a plate with an elongated hole that the meniscus was pulled against thereby loading the collagen bundle only. A novel test device shown in (**b**) and (**c**) was constructed to mimic physiological load distribution on menisci, which are still attached to the tibial bone. Hemispheres were chosen according to the meniscus’ size and pulled down for load application. **d** shows a meniscus still attached to a bone block. The distance between the inner meniscal vertex and the bone block is referred to as L_0_. The width of the bone block is 12 mm. **e** Equal anterior (h_a_) and posterior (h_b_) heights of the bone block were ensured during the preparation process

All allografts were provided by a non-profit tissue bank (German Institute for Cell and Tissue Replacement, DIZG gemeinnützige GmbH). All tissues are acquired from non-profit tissue recovery partners after informed consent. Sterilisation was performed using a validated, GMP-compliant process. Sterile allografts are approved as medicinal products under §21 of the German *Medicinal Products Act* (license number: PEI.H.03360.01.1). Knees from a single donor are thawed at 2–8 °C and menisci are exposed and visually evaluated after removing remnants of blood, fat, muscles and connective tissue. Previously injured menisci or knees showing signs of severe cartilage degeneration were excluded. The tibial plateau was cut 10–15 mm distal to the articular surface using a band saw. One of the two menisci was cut close to the root attachments for further determination of suture retention and histology leaving the other still attached to the tibial plateau. As depicted in Fig. 1e the plateau was cut to 12 mm width and maximum 10 mm anterior height without compromising the meniscal structure. The posterior as well as the contralateral bone blocks were adjusted to equal heights. A total of 31 menisci pairs from 26 male and 5 female donors were included in this analysis. The average donor age was 49.87 years and ranged from 32 to 65 years. For the different testing parameters, the menisci were all analysed pairwise.

### Peracetic acid-ethanol sterilisation

All menisci pairs were thawed in their packaging and placed in containers filled with sterile *physiological sa*line solution. Menisci were left to thaw for at least 30 min at room temperature. Once thawed one underwent sterilisation, the other was kept at room temperature. Both groups contained almost equal numbers of right and left menisci to account for side specific differences. For sterilisation, tissues were fully submerged in validated tissue-preserving sterilisation solution (2% peracetic acid, 96% ethanol, aqua ad iniectabilia; ratio v/v/v | 2/1/1) and incubated under constant agitation, at low pressure and at room temperature for 4 h. Subsequently, tissues were rinsed in a washing process using aqua ad iniectabilia. All menisci were stored at − 30 °C for further analysis.

### Suture retention

Suture retention tests were performed with 13 pairs of menisci (mean age 50.38 years, range 37–63). Suture loops were placed around equal meniscus cross-sections (0.5 mm^2^) using a 0.6 mm suture material (see Fig. 1a). The collagen bundles were pulled to failure through an elongated hole providing a barrier for the rest of the meniscus as previously performed with a collagen meniscus implant (CMI) [17]. A 200 N ElectroForce® TestBench (Bose, USA) was used to determine stiffness and failure load (preload: 10 N, speed: 0.05 mm/s).

### Physiological testing

Pairwise physiological testing was performed with 22 pairs of menisci (mean age 50.59 years, range 32–65). A total of 14 medial and 8 lateral menisci pairs were tested pairwise to their counterparts. A newly developed test device (Fig. 1b and c) was constructed to mimic physiological load distribution on the femoral condyle contact area. Prior to the mechanical testing, the menisci were thawed at 35 ± 2 °C for at least 15 min followed by measuring the distance between meniscal vertex and the bone block (L_0_) using a caliper (Fig. 1d). Bone blocks were then fixed and hemispheres of matched size were placed between the meniscal vertex and the bone block. Mechanical tests were performed at 35 ± 2 °C in 0.9% saline solution. Temperature was confirmed using a Hamster data logger (Elpro, Germany).

Mechanical tests were conducted in three stages using a Zwick Z010 test machine (Zwick, Germany). First, the menisci were loaded to 200 N at 2%/s applying a preload of 5 N. The crossbar’s positions at 5 and 200 N were recorded and used as the lower and upper limit during preconditioning, respectively. Menisci were preconditioned 9 times at 0.1 Hz with the load-time-graph following a cosine function. At the peak of the 10th cycle, the position was held constant for 45 min. Load relaxation was determined.

After a recovery phase at 4 °C in 0.9% saline solution over night, 8 out of 22 meniscus pairs (men age 44 years, range 32–64) underwent a 10 cycle preconditioning according to day 1 and were tested again for their relaxation behaviour on 5 consecutive days. An Inspekt table blue 10 kN machine (Hegewald & Peschke, Germany) was used to perform repetitive tests. One to three days after relaxation testing, menisci were loaded to failure following a 10 cycle preconditioning similar to day 1. Stiffness and load to failure were assessed.

### Moisture content

Moisture content of 18 menisci pairs composed of 9 medial and 9 lateral pairs (mean age 54.88 years, range 43–65) was evaluated by thermogravimetric analysis using the HX204 Moisture Analyser (Mettler Toledo, Germany). Similar segments of meniscus pairs were dissected resulting in 0.2 to 0.8 g specimens. Specimens were gently dabbed prior to the measurement using a lint-free tissue. After preheating the analyser to 100 °C, moisture content was assessed using a standard drying program, a temperature of 105 °C and a constant weight criterion of 0.1%/5 min.

### Histological analysis

Meniscus segments of equal size and origin of 2 pairs of menisci (*n* = 4) were sent for histological evaluation (Morphisto GmbH, Germany). Briefly, fixed tissues were dehydrated and paraffin embedded. Longitudinal and cross sections of 5 μm thickness were hematoxylin and eosin (H&E) stained and mounted on slides. Microscopic analysis was performed using a DMIL microscope (Leica, Germany). Micrographs of similar zones were compared with regard to their general appearance and the presence of interspaces (lacunae).

### Statistical analysis

Data are presented as *mean* ± standard error of *mean* (*SEM*). Statistical significance was set with *p* <  0.05. Gaussian distributions were confirmed by D’Agostino-Pearson tests and the data were analysed using paired student’s t-tests. (*: *p* ≤ 0.05; **: *p* ≤ 0.01; ***: *p* ≤ 0.001; ****: *p* ≤ 0.0001). Unless stated otherwise significant differences between the two groups are shown in brackets.

## Results

### Suture retention analysis

**Fig. 2:**
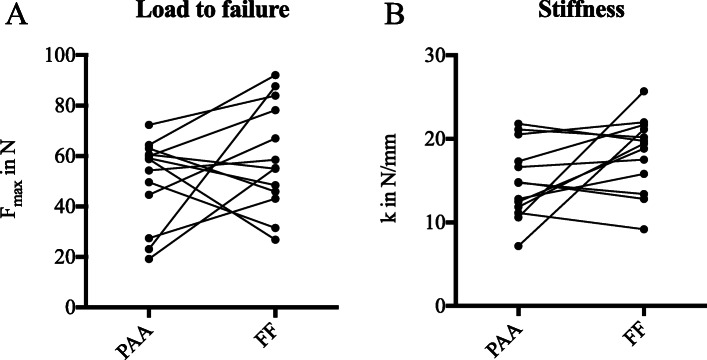
Stiffness (**a**) and load to failure (**b**) determined by suture retention analysis of 13 pairs of menisci. No significant difference between peracetic acid (PAA) sterilised and fresh-frozen (FF) menisci could be demonstrated with regards to stiffness and load to failure

Pairwise comparison of 13 meniscus pairs revealed no significant difference between PAA sterilised (14.85 ± 4.46 N/mm, 50.49 ± 17.01 N) and fresh-frozen (18.26 ± 4.46 N/mm, 59.49 ± 21.07 N) menisci regarding stiffness and load to failure, respectively (Fig. 2).

### Physiological testing

**Fig. 3:**
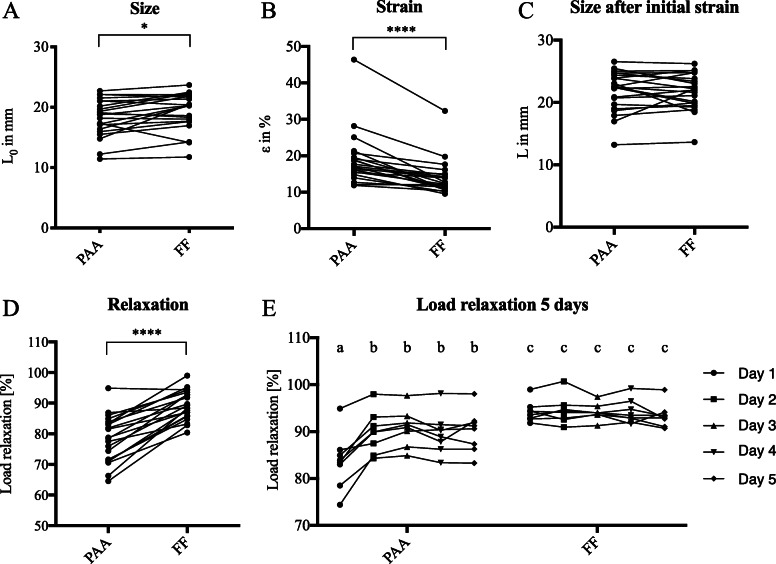
Twenty two pairs of menisci of which one meniscus was PAA treated and the other was left untreated (FF), were biomechanically analysed using a novel meniscus fixture. Prior to the testing, meniscus length was measured (**a**). Strain was measured and meniscal size was calculated after the application of a 200 N load (**b** and **c**). While keeping the strain constant for 45 min, the tissue starts to relax (**d**). On a subset of 8 menisci this testing was performed in 5 consecutive days (**e**). Two groups sharing the same character are not significantly different (*p* < 0.05)

Using a novel custom built test device that allows the application of physiological loads, 22 pairs of menisci were tested for viscoelastic and failure properties. Prior to biomechanical evaluation, meniscus size (L_0_) was determined. Menisci showed a significant size reduction after treatment with peracetic acid (18.30 ± 0.66 for PAA compared to FF 19.39 ± 0.65, *p*-value 0.0126) (Fig. 3a). Strain measurements at 200 N revealed a significantly higher strain for PAA (18.87 ± 1.56) treated menisci in comparison to the untreated controls (13.81 ± 1.04, p-value < 0.0001) (Fig. 3b). After the application of 200 N the menisci in the two groups displayed similar sizes, PAA (21.87 ± 0.69) and FF (21.73 ± 0.62) *p*-value 0.7360 (Fig. 3c). For detailed results see Table 1.

**Table 1:** Results of physiological and biomechanical comparison of peracetic acid sterilised (PAA) versus untreated fresh frozen (FF) menisci. Twenty two meniscus pairs were examined with regards to size, strain and size at 200 N, relaxation properties as well as stiffness and load to failure. Values are presented as MEAN ± SEM

	Size in mm	Strain in %	Size at 200 N in mm	Relaxation in %	Stiffness in N/mm	Load to failure in N
PAA	18.30 ± 0.66	18.87 ± 1.56	21.87 ± 0.69	77.71 ± 1.62	195.0 ± 13.1	700.1 ± 71.4
FF	19.39 ± 0.65	13.81 ± 1.04	21.73 ± 0.62	89.11 ± 1.00	192.9 ± 11.8	770.1 ± 76.2
p-value	0.0126	< 0.0001	0.7360	< 0.0001	0.8587	0.3093

**Fig. 4:**
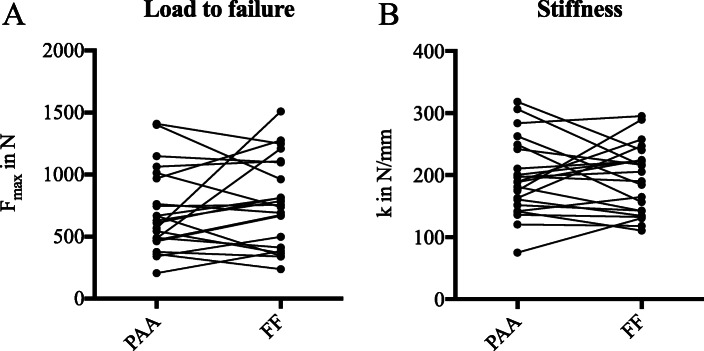
Meniscus pairs of 22 donors were analysed for failure properties. Menisci that were still attached to a tibial bone block, were fixed in a novel device to mimic physiological load distribution. Following 10 preconditioning cycles, stiffness (**a**) and load to failure (**b**) were determined comparing PAA sterilised menisci to their untreated controls

Relaxation testing assessed viscoelastic properties. Lower relaxation was observed for PAA treated menisci at day 1 of physiological testing (Fig. 3d). Additional testing of a subgroup of 8 menisci on 5 consecutive days confirmed the significant differences in relaxation, PAA (77.71 ± 1.62) and FF (89.11 ± 1.00) p-value < 0.0001 (Fig. 3e). Furthermore, PAA treated menisci showed a significantly lower relaxation on day 1 compared to the following days. No significant differences in either stiffness or failure load were demonstrated using the physiological test setup (Fig. 4). Here, failure always occurred through bony avulsion.

### Moisture content

Moisture content was determined thermogravimetrically. PAA-sterilisation was found to lower the moisture content from 72.74 ± 2.56% to 68.47 ± 2.32% (*p* <  0.0001).

### Histological analysis

**Fig. 5:**
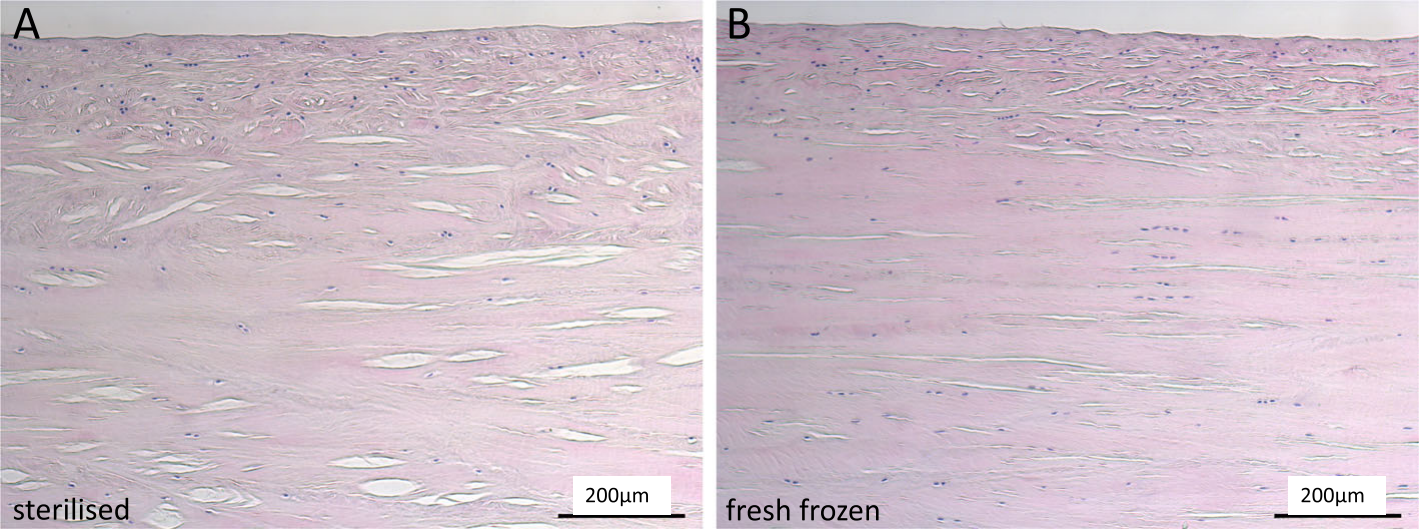
Representative longitudinal sections of PAA treated (**a**) and fresh-frozen (**b**) menisci are shown after H&E staining. The scale bar depicts 200 μm and *n* = 4. Evenly distributed lacunae were present in both types of menisci. Lacunae were wider in PAA treated tissue

H&E stained histological sections of paraffin embedded samples showed widened lacunae in PAA treated menisci (Fig. 5).

## Discussion

The meniscus has many biomechanical roles that vary from load transmission [53], stability [29, 52], joint lubrication [46], reduction of contact stress by providing an increased contact area [25, 58] and knee congruity [58]. Various factors can affect MAT performance, some examples are graft size, fixation technique, level of activity and age [1, 4, 11, 32, 44]. Analysis into different biomechanical parameters comparing PAA sterilised and non-sterilised menisci were conducted. In the present study, a biomechanical equivalence between PAA sterilised and non-sterilised menisci with regards to stiffness and load to failure in the two different test setups was demonstrated. Furthermore, suture retention tests are normally used to describe optimal meniscus repair techniques. Referring to root attachment fixation, sutures are loaded alongside the collagen fibers, leading to failure loads lower than 200 N [26, 35]. Here, the load was applied perpendicularly to the fiber’s orientation. Pretests (unpublished data) only showed suture failures after testing entire meniscus cross sections. Therefore, partial cross sections (0.5 mm^2^) were tested. Suture fixation is an important biomechanical parameter in successful meniscal repair and is required to withstand the different loads applied [39]. Failure loads of longitudinal repair techniques vary widely in the literature ranging from 57.8 to 161 N based on the experimental set up [2, 7, 31, 50]. The load to failure values presented here (PAA sterilised 50.49 ± 17.01 N and fresh-frozen 59.49 ± 21.07 N) are at the lower end compared to the values documented in the literature. However, differences in experimental set ups were observed and varied from porcine, sheep and calf models. The differences in influencing factors such as properties of the suture material, area of the sutured meniscal cross section and the mechanical test procedure itself complicate a thorough comparison of the suture retention results. Stiffness measurements conducted here displayed values of 14.85 ± 4.46 N/mm for PAA and 18.26 ± 4.46 N/mm for fresh- frozen allografts, similar to that reported in the literature ranging from (2.2–67.8 N/mm) [7, 31, 50].

Additional biomechanical testing was conducted and investigated the stiffness and load to failure of menisci. These parameters are of importance as stiffness is defined as the resistance to deformation under applied forces and is important for successful MAT [5]. Consistent with the suture retention results, stiffness and load to failure were not significantly different between PAA treated and untreated menisci using the novel physiological test setup. In 2010, Hauch et al. determined the failure properties of human meniscus root attachments showing slightly lower values for average load to failure of 500.6 ± 232.5 N and an almost identical stiffness of 180.5 ± 59.5 N/mm [23]. The group tested one root attachment at a time, whereas in the present study tensile stress was applied on both root attachments. Ellman et al. also investigated single meniscal root attachments biomechanically [12]. Ellman pointed out the role of tissue preparation. Resection of supplemental fibers has a negative effect on the failure properties of meniscal attachments. Native roots were tested with ultimate failure loads ranging from approximately 500 to 660 N. According to the observations of both Ellman and Hauch failure always occurred through bony avulsion as observed here in this study.

In 2005, Scheffler and colleagues tested PAA sterilised human BPTB grafts. No significant differences in stiffness and load to failure were detected between sterilised BPTBs and the untreated counterparts [51]. In contrast to the failure properties, evaluation of time dependent biomechanical properties showed an effect of PAA treatment. On the one hand, sterilised menisci were smaller than the untreated ones but on the other hand they stretched more when subjected to the same load of 200 N. The higher initial strain compensates for the size reduction. After calculation of the size of the menisci at the first load peak, there are no significant differences between PAA and untreated menisci indicating preserved structural integrity of PAA menisci.

Sterilised menisci relaxed less compared to fresh frozen menisci. This was confirmed on five consecutive days. According to Hauch and colleagues the relaxation could affect the position of the meniscus graft within the joint [23]. Meniscus extrusion could be enhanced in meniscal replacements showing high relaxation. Thus, meniscal extrusion is associated with knee malalignment, cartilage damage and meniscal tears [10]. An extrusion of more than 3 mm is hypothesized to facilitate biomechanical changes and osteoarthritis [8–10, 30, 37]. PAA treated menisci show less load relaxation after a 10 cycle preconditioning phase, suggesting superior extrusion properties compared to untreated menisci.

The *meniscus*, an extracellular matrix is mainly composed of water. It contains approximately 70% water (liquid phase) and a 30% solid phase. The solid phase mainly consists of collagenous proteins which are accompanied by a low percentage of proteoglycans such as glycosaminoglycans, which are key players with regards to viscoelastic properties of the tissue [3, 17]. These viscoelastic properties depend on the biphasic composition of the meniscal tissue [14]. They have water retention capability and high resistance to compressive loads [3, 18, 40]. Therefore, the moisture content was investigated with both lateral and medial menisci. In this study, the water content of PAA treated menisci was significantly lower compared to untreated menisci, which can account for the significantly lower and slower relaxation of PAA treated menisci revealed by relaxation tests.

A degradation of proteoglycans can explain both, the lower residual moisture and the altered viscoelastic properties of PAA sterilised menisci. However, 5% PAA does not reduce the glycosaminoglycan content of tendons [57].

The reduced water content might also be a consequence of the macroscopic structural changes seen in PAA sterilised tissues. In 2011, Woon et al. treated acellularized intrasynovial tendons with 5% PAA for 4 h to enhance reseeding capability. They too observed larger interspaces between the collagen bundles. PAA treated tendons showed greater reseeding, no cytotoxicity and were not inferior to untreated controls with regards to ultimate tensile stress and elastic modulus [59]. Vascular access channels generated by trephination are described as meniscal repair facilitators [3, 18]. The interspaces between collagen bundles of sterilised menisci found in this study might accelerate the reseeding process as they facilitate cell-to-tissue contact.

In contrast, Scheffler et al. performed biomechanical tests with PAA sterilised anterior cruciate ligament (ACL) constructs 6 and 12 weeks after transplantation into sheep. PAA treated ACL grafts were significantly weaker and less stiff [50]. In contrast to the menisci used in the present study, the tendons used in the Scheffler study were delipidised with chloroform, methanol and ultrasound.

The present study has certain limitations. Here, the physiological test device only accounted for one major meniscus transplantation technique, the bone block fixation but did not include other methods such as the soft tissue technique. Also, meniscus grafts will certainly be transplanted using the keyhole technique too. Aged related changes to the meniscus have been previously reported [55]. This study also included mensicus harvested from aged donors, however the pairwise comparisons conducted here have minimized any potential bias. Additionally the biomechanical testing conducted here cannot be directly compared to clinical success and would need to be investigated further. Differences in experiemental set up used here and in the literature make it difficult to directly compare this work. The present study is experimental and the positive effects of PAA sterilised menisci regarding meniscal extrusion would need to be clinically verified.

## Conclusion

Human PAA sterilised menisci performed equally to fresh-frozen counterparts in a suture retention test and in physiological failure testing. Meniscus relaxation, biphasic properties and strain were shown to be significantly different between the two groups. Treated menisci displayed lower relaxation and moisture content and higher strain compared to untreated menisci. PAA sterilised menisci might exhibit properties positively influencing meniscus extrusion after transplantation.

## Abbreviations

ACL: Anterior Cruciate Ligament
BPTB: Bone - Patellar Tendon – Bone
CMI: Collagen Meniscus Implant
FF: Fresh-Frozen
H&E: Hematoxylin and eosin
PAA: Peracetic Acid
